# Clinicopathological Predictors of Positive Resection Margins in Breast-Conserving Surgery

**DOI:** 10.1245/s10434-024-15153-8

**Published:** 2024-03-23

**Authors:** Hemali Chauhan, Natasha Jiwa, Vikneswaran Raj Nagarajan, Paul Thiruchelvam, Katy Hogben, Ragheed Al-Mufti, Dimitri Hadjiminas, Sami Shousha, Ramsey Cutress, Hutan Ashrafian, Zoltan Takats, Daniel Richard Leff

**Affiliations:** 1 Department of Surgery and Cancer, Imperial College London, London, UK; 2 Breast Unit, Charing Cross Hospital, Imperial College NHS Trust, London, UK; 3 North West London Pathology, Imperial College NHS Trust, London, UK; 4 Faculty of Medicine, University of Southampton, Southampton, UK

**Keywords:** Breast-conserving surgery, Positive margin predictors

## Abstract

**Background:**

Ductal carcinoma in situ (DCIS) is associated with risk of positive resection margins following breast-conserving surgery (BCS) and subsequent reoperation. Prior reports grossly underestimate the risk of margin positivity with IBC containing a DCIS component (IBC + DCIS) due to patient-level rather than margin-level analysis.

**Objective:**

The aim of this study was to delineate the relative risk of IBC + DCIS compared with pure IBC (without a DCIS component) on margin positivity through detailed margin-level interrogation.

**Methods:**

A single institution, retrospective, observational cohort study was conducted in which pathology databases were evaluated to identify patients who underwent BCS over 5 years (2014–2019). Margin-level interrogation included granular detail into the extent, pathological subtype and grade of disease at each resection margin. Predictors of a positive margin were computed using multivariate regression analysis.

**Results:**

Clinicopathological details were examined from 5454 margins from 909 women. The relative risk of a positive margin with IBC + DCIS versus pure IBC was 8.76 (95% confidence interval [CI] 6.64–11.56) applying UK Association of Breast Surgery guidelines, and 8.44 (95% CI 6.57–10.84) applying the Society of Surgical Oncology/American Society for Radiation Oncology guidelines. Independent predictors of margin positivity included younger patient age (0.033, 95% CI 0.006–0.060), lower specimen weight (0.045, 95% CI 0.020–0.069), multifocality (0.256, 95% CI 0.137–0.376), lymphovascular invasion (0.138, 95% CI 0.068–0.208) and comedonecrosis (0.113, 95% CI 0.040–0.185).

**Conclusions:**

Compared with pure IBC, the relative risk of a positive margin with IBC + DCIS is approximately ninefold, significantly higher than prior estimates. This margin-level methodology is believed to represent the impact of DCIS more accurately on margin positivity in IBC.

**Supplementary Information:**

The online version contains supplementary material available at 10.1245/s10434-024-15153-8.

Breast-conserving surgery (BCS) is the most common surgical treatment for early invasive breast cancer (IBC) and ductal carcinoma in situ (DCIS) in the United Kingdom (UK) and United States (US).^1–4^ In the UK, a recent ‘Getting It Right First Time’ report estimated 70% of patients undergo BCS as their first surgery for breast cancer.^4^ BCS has many advantages over mastectomy, but is complicated by a risk of involved resection margins leading to reoperation in approximately 17–33% of patients.^4–10^ The national mean re-excision rate in the UK was 18.8% between 2015 and 2018.^4^ Following the introduction of a best practice toolbox, re-excision rates approximately halved, from 20–24% to 12.3% in the US.^7,11–13^ Reoperative intervention is associated with significant human and economic burden.^14,15^ Reoperation increases the risk of postoperative complications, inferior patient satisfaction and cosmetic results, and a greater burden on health services.^14,16–19^

It is widely documented that the presence of DCIS increases the likelihood of a positive margin, due to the impalpable nature of the disease, the unpredictable extension of DCIS beyond the edge of a palpable invasive tumour, and the lack of specific clinical correlates leading to radiological underestimates;^20–22^ however, quantifying the exact impact of DCIS on positive margin rates is less well documented. Rates of positive margins associated with pure DCIS reported in the literature range between 30 and 63%,^5,21–29^ compared with 14–27% for invasive disease.^5,21,22,25^ In the UK, data from Hospital Episode Statistics suggest rates of reoperative intervention for close-positive margins following failed BCS were substantially higher for pure DCIS (29.5%) versus invasive disease with or without a DCIS component (18%).^5^ Moreover, DCIS has been observed to be an independent predictor of positive resection margins in many published series.^30–36^ However, we suspect that the magnitude of positive margins associated with DCIS may have been underreported, since prior studies employed patient-level core biopsy data (DCIS present in the diagnostic biopsy vs. no DCIS present in the diagnostic biopsy)^30–32,34,36–40^ or preclassified patient-level data held in National databases (DCIS component in the primary tumour vs. no DCIS component in the primary tumour)^5,7,21,33,35^ rather than a detailed margin-level analysis (DCIS present at the margin vs. no DCIS present at the margin) to characterise and validate the histopathological relationships.

In this study, we aimed to overcome the limitations of prior work through detailed margin-level analysis, using histopathology specimen reports to extract the extent, pathological entity and grade of disease responsible for each positive margin. Moreover, we sought to determine the association between clinicopathological variables, including the presence of DCIS and positive margins. To our knowledge, there have been no prior studies utilising margin-level data to calculate these associations. Arguably, this level of methodological detail will improve the clinical utility of any associations between clinicopathological variable and margin positivity associated with a DCIS component. This may inform perioperative patient counselling and surgical strategy for BCS and ultimately reduce the burden of reoperative breast surgery.

## Methods

### Overview

A single institution, retrospective, observational cohort study was conducted in which a pathology database was interrogated to identify patients who underwent BCS at our institution between 1 December 2014 and 31 November 2019. This evaluation included all women aged over 16 years undergoing BCS for IBC and/or DCIS, and excluded those who received neoadjuvant chemotherapy (NAC). NAC can downstage tumours and reduce tumour size, which can alter margin status and skew the results from this study.^41^ The study was registered as a service evaluation with the Data and Intelligence Department at Imperial College Healthcare NHS Trust (ID = SPS_032).

### Positive Margin Definition

Margin status was the main outcome measure; cases were coded based on margin status (negative = 0, positive = 1). Margin status was defined as per UK Association of Breast Surgery (ABS) guidelines,^42^ i.e. invasive or in situ disease <1 mm from the inked resection margin. This guideline, employed at our institution, is the most commonly used guideline for acceptable margin width in the UK.^43^ In addition, Society of Surgical Oncology/American Society for Radiation Oncology (SSO-ASTRO) guidelines^44^ were applied to the dataset, in which a positive margin is defined as ‘tumour on inked margin’ for IBC and/or presence of DCIS < 2 mm from the inked resection margin.^45^ This parallel approach allowed comparisons in potential reoperation rates between the UK and US in the same BCS cohort. For the purposes of this project, a positive margin (PM) refers to a positive radial margin i.e. superior, inferior, medial or lateral.

### Data Collection and Analysis

Hospital databases combine inpatient and outpatient clinical documentation, multidisciplinary team meeting outcomes, and results and reports of investigations into an electronic patient record (Cerner^®^). Similarly, a dedicated database was used by histopathologists for entering and storing pathology reports (CoPath^®^). The electronic patient record and pathology database were used to collect data on several clinicopathological variables for each patient.

More than 100 data points were recorded for each patient, including sociodemographic variables: patient age and referral route, i.e. symptomatic or screening recall. Preoperative data were recorded, including core biopsy date; core biopsy histopathology findings (e.g. IBC, DCIS, mixed IBC and DCIS); imaging features, including presence or absence of microcalcifications; breast density (not dense = Breast Imaging Reporting and Data System [BI-RADS] A, B/dense = BI-RADS C, D);^46^ and whether an MRI was carried out (yes/no). Primary surgery details included date of surgery; whether localisation was performed (yes/no); specimen weight (g); tumour type (e.g. invasive, ductal, lobular, etc.) and grade (I–III); tumour size (mm); multifocality (yes/no); hormone receptor status (estrogen receptor [ER], progesterone receptor [PR], human epidermal growth factor receptor 2 [HER2] positive/negative); the presence of DCIS and its grade (low grade [LG]/intermediate grade [IG]/high grade [HG]); lymphovascular invasion (LVI; present or not); and/or comedonecrosis (present or not).

Intraoperative data collected included whether additional shaves were taken (number of additional shaves) and the margin shave histology (tumour type [e.g. invasive ductal, lobular, etc.] and grade [I–III], tumour size [mm], multifocality [yes/no], hormone receptor status [ER, PR, HER2 positive/negative], and the presence of DCIS and its grade [LG/IG/HG]). Margin-level data included margin positivity (yes/no); location of positive margin(s) [anterior, posterior, superior, inferior, medial, lateral]; pathological entity responsible for each positive margin, including tumour distance from margin (mm); tumour type (e.g. invasive, ductal, lobular, etc.) and grade (I–III); tumour size (mm); multifocality (yes/no); the presence of DCIS and its grade (LG/IG/HG); and comedonecrosis (present or not).

Reoperation data incorporated the date of reoperation, type of reoperation (re-excision of margins/mastectomy), reoperation histology (tumour type [e.g. invasive ductal, lobular, etc.] and grade [I–III]), tumour size (mm), multifocality (yes/no), the presence of DCIS and its grade (LG/IG/HG), reoperation margin positivity (yes/no), location of positive margin(s) [anterior, posterior, superior, inferior, medial, lateral], details of the pathological entity responsible for each positive reoperation margin, including tumour distance from margin (mm), tumour type (e.g. invasive, ductal, lobular, etc.) and grade (I–III), tumour size (mm), multifocality (yes/no), and the presence and grade of DCIS (LG/IG/HG).

Statistical analysis was performed using SPSS version 28 (IBM Corporation, Armonk, NY, USA). Using transparent reporting of a multivariable prediction model for individual prognosis or diagnosis (TRIPOD) guidelines (48), univariate and multivariate analysis was performed using logistic regression to identify clinicopathological predictors of positive margin and reoperation.^47,48^ Chi-square tests were carried out for categorical data. A *p* value < 0.05 was presumed to be statistically significant. There were no missing data in the final dataset as all data had been individually extracted from hospital databases.

## Results

### Positive Margin Rate

**Fig. 1:**
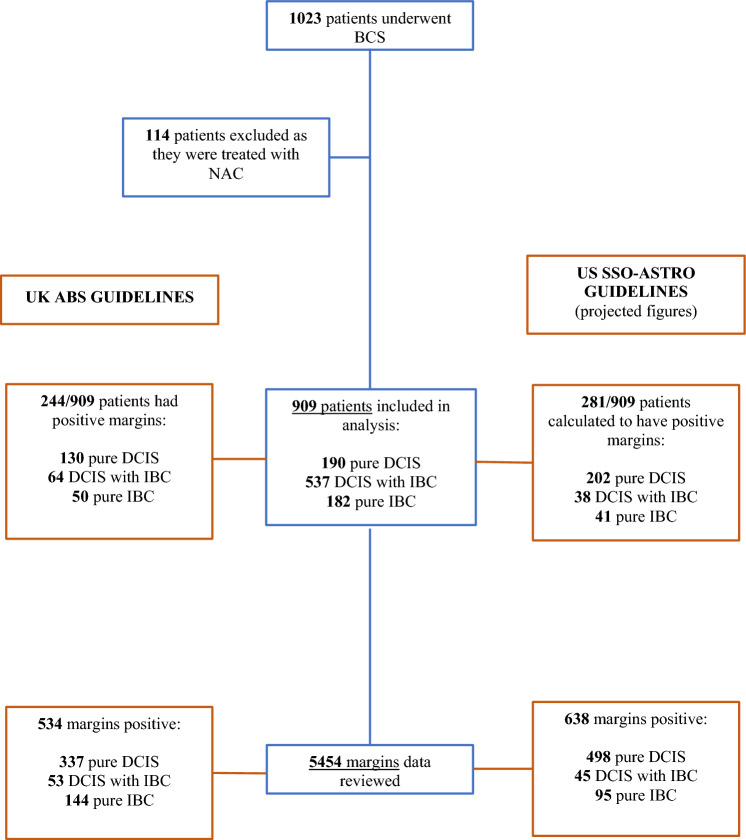
Study flow of patients using margin-level data, detailing exclusion criteria and positive margin rates with histology breakdown based on total patient number (*n* = 909) or total margin number (*n* = 5454) comparing UK ABS guidelines with US SSO-ASTRO projected figures. *ABS* Association of Breast Surgery, *ASTRO* American Society for Radiation Oncology, *BCS* breast-conserving surgery, *DCIS* ductal carcinoma in situ, *IBC* invasive breast cancer, *NAC* neoadjuvant chemotherapy, *SSO* Society of Surgical Oncology

Between 1 December 2014 and 31 November 2019, 1023 patients underwent BCS at our institution; 114 women underwent NAC and were thus excluded. Clinicopathological details from the remaining 909 patients were examined, equating to 5454 margins (further clinicopathological data results can be found in Online Resource Table 1). Applying the ABS guidelines, 27% (244/909) of patients had a positive margin and 26% (239/909) underwent reoperation. Applying SSO-ASTRO guidelines would have increased the positive margin rate to 31% (281/909), as demonstrated in Fig. 1.

### Ductal Carcinoma In Situ-Associated Margin Positivity

Margin-level data were reviewed for 5454 individual margins. According to ABS guidelines, 686/5454 margins were positive, of which 534/686 were positive margins. Correspondingly applying SSO-ASTRO guidelines, 770/5454 would have been judged positive margins and 638/770 were positive margins.

Pure DCIS was the most common cause of radial margin positivity [337/534 (63%), 498/638 (78%)] compared with mixed DCIS and IBC [53/534 (10%), 45/638 (7%)] or pure IBC [144/534 (27%), 95/638 (15%)] (ABS, SSO-ASTRO criteria, respectively; a full breakdown of positive margin data is shown in Online Resource Table 2). The relative risk (RR) of finding DCIS at the margin (pure DCIS or DCIS with IBC) versus pure IBC at the margin was 8.76 (95% confidence interval [CI] 6.64–11.56) when applying ABS guidelines, and 8.44 (95% CI 6.57–10.84) for SSO-ASTRO guidelines (see Table 1). Of the positive margins with a DCIS component, HG DCIS was more commonly present (48%, 48%) compared with IG DCIS (24%, 36%,) or LG DCIS (18%, 16%) (ABS, SSO-ASTRO criteria, respectively).

**Table 1:** The RR of DCIS at the margin versus invasive breast cancer at the margin in patients with a positive margin

Histology	Margin [*n* (%)]		Primary tumour [*n* (%)]		Biopsy [*n* (%)]	
	ABS	SSO-ASTRO	ABS	SSO-ASTRO	ABS	SSO-ASTRO
IBC + DCIS	194/244, 80%	214/281, 76%	199/244, 82%	251/281, 89%	99/244, 41%	132/281, 47%
Pure IBC	50/244, 20%	67/281, 24%	45/244, 18%	30/281, 11%	145/244, 29%	149/281, 53%
RR (95% CI)	8.76 (6.64–11.56)	8.44 (6.57–10.84)	1.46 (1.10–1.95)	2.77 (1.96–3.92)	1.19 (0.99–1.43)	1.52 (1.28–1.80)

The RRs are calculated at margin level (DCIS present at the margin vs. no DCIS present at the margin), patient level (DCIS component in the primary tumour vs. no DCIS component in the primary tumour) and preoperatively (DCIS present in the diagnostic biopsy vs. no DCIS present in the diagnostic biopsy)

*ABS* Association of Breast Surgery, *ASTRO* American Society for Radiation Oncology, *CI* confidence interval, *DCIS* ductal carcinoma in situ, *IBC* invasive breast cancer, *RR* relative risk, *SSO* Society of Surgical Oncology

There was a statistical association between the presence of DCIS at a positive margin and younger patient age (*p *= 0.015), lower specimen weight (*p *= 0.006), impalpable tumours (wire-guided surgery; *p *= 0.029), multifocal disease (*p *< 0.001), fewer intraoperative shaves (*p *= 0.001), the presence of microcalcifications (*p *< 0.001), LVI (*p *= 0.002), comedonecrosis (*p *< 0.001), and higher-grade DCIS (*p *< 0.001) (Table 2).

**Table 2:** Chi-square cross-tabulation results assessing the relationship between various categorical clinicopathological variables and the presence of DCIS at a positive margin

Variable	UK: ABS guidelines		US: SSO-ASTRO guidelines	
	Chi-square value	*p* value	Chi-square value	*p* value
Younger age	5.206	0.285	12.296	0.015
Lower specimen weight	14.545	0.006	3.987	4.08
Wire-guided surgery	1.319	0.251	4.796	0.029
Microcalcifications	30.316	< 0.001	17.834	< 0.001
Multifocal disease	12.966	< 0.001	12.966	< 0.001
Lymphovascular invasion	7.347	0.007	9.448	0.002
Comedonecrosis	95.747	< 0.001	64.826	< 0.001
Higher DCIS grade	128.036	< 0.001	111.038	< 0.001
Fewer intraoperative shaves	6.464	0.011	10.94	0.001

*ABS* Association of Breast Surgery, *ASTRO* American Society for Radiation Oncology, *DCIS* ductal carcinoma in situ, *SSO* Society of Surgical Oncology

### Patient-Level Data

Of the 244 patients with a positive margin according to ABS guidelines, a DCIS component was identified in 184/244 (75.4%). Specifically, 124/244 (50.8%) patients demonstrated pure DCIS disease, 60/244 (24.6%) demonstrated mixed invasive and DCIS disease, and 60/244 (24.6%) demonstrated pure IBC. Applying the SSO-ASTRO guidelines increased the proportion of patients with a DCIS component within their primary tumour to 240/281 (85.4%), of whom 202/281 (84.2%) had pure DCIS and 38/281 (15.8%) had mixed invasive and DCIS disease.

### Predictors of a Positive Margin

Univariate analysis based on ABS criteria demonstrated younger age at index operation (0.035, 95% CI 0.007–0.062; *p *= 0.015), lower specimen weight (0.030, 95% CI 0.005–0.055; *p *= 0.019), multifocality (0.256, 95% CI 0.134–0.378; *p *< 0.001), mammographic microcalcifications (0.080, 95% CI 0.021–0.138; *p *= 0.007), HER2-positive status (0.380, 95% CI 0.003–0.073; *p *= 0.034), larger composite tumour size (0.012, 95% CI 0.010–0.014; *p *< 0.001), DCIS present beyond IBC boundaries (0.139, 95% CI 0.082–0.195; *p *< 0.001), the presence of DCIS (0.151, 95% CI 0.080–0.223; *p *< 0.001), DCIS with comedonecrosis (0.182, 95% CI 0.114–0.025; *p* < 0.001) and LVI (0.137, 95% CI 0.067–0.206; *p *< 0.001) were significantly associated with a positive margin.

Upon computing the univariate associations between clinicopathological variables and positive margin using SSO-ASTRO criteria, similar associations were observed. Specifically, younger age at index operation (0.054, 95% CI 0.025–0.083; *p *< 0.001), the presence of mammographic microcalcifications (0.122, 95% CI 0.061–0.182; *p *< 0.001), multifocal disease (0.193, 95% CI 0.065–0.321; *p *= 0.003), tumour grade (0.040, 95% CI 0.008–0.072; *p *= 0.013), ER-positive status (0.123, 95% CI 0.061–0.186; *p *< 0.001), PR-positive status (0.090, 95% CI 0.041–0.140; *p *< 0.001), HER2-positive status (0.940, 95% CI 0.058–0.131; *p *< 0.001), larger tumour size (0.011, 95% CI 0.009–0.014; *p *< 0.001), DCIS present beyond IBC boundaries (0.240, 95% CI 0.183–0.298; *p *< 0.001), the presence of LVI (0.110, 95% CI 0.038–0.182; *p *= 0.003), the presence of DCIS (0.243, 95% CI 0.170–0.317; *p *< 0.001), DCIS with comedonecrosis (0.260, 95% CI 0.190–0.330; *p *< 0.001) and fewer immediate shave margins (0.092, 95% CI 0.031–0.152; *p *= 0.003) were associated with a positive margin (Table 3).

**Table 3:** Univariate analysis of association with positive margins comparing UK ABS guidelines with US SSO-ASTRO guidelines

Variable	UK ABS		US SSO-ASTRO	
	*β* coefficient (95% CI)	*p* value	*β* coefficient (95% CI)	*p* value
Younger age at index operation, years	0.035 (0.007–0.062)	0.015	0.054 (0.025–0.083)	< 0.001
Lower specimen weight, g	0.03 (0.005–0.055)	0.019	–	–
Mammographic microcalcifications present	0.08 (0.021–0.138)	0.007	0.122 (0.061–0.182)	< 0.001
Multifocal disease	0.256 (0.134–0.378)	< 0.001	0.193 (0.065–0.321)	0.003
Tumour grade	–	–	0.04 (0.008–0.072)	0.013
ER-positive status	–	–	0.123 (0.061–0.186)	< 0.001
PR-positive status	–	–	0.09 (0.041–0.14)	< 0.001
HER2-positive status	0.38 (0.003–0.073)	0.034	0.94 (0.058–0.131)	< 0.001
Larger composite size of tumour	0.012 (0.01–0.014)	< 0.001	0.011 (0.009–0.014)	< 0.001
DCIS present beyond invasive disease?	0.139 (0.082–0.195)	< 0.001	0.24 (0.183–0.298)	< 0.001
Lymphovascular invasion present	0.137 (0.067–0.206)	< 0.001	0.11 (0.038–0.182)	0.003
DCIS with comedonecrosis	0.182 (0.114–0.25)	< 0.001	0.26 (0.19–0.33)	< 0.001
DCIS present	0.151 (0.08–0.223)	< 0.001	0.243 (0.170–0.317)	< 0.001
Fewer shaves	–	–	0.092 (0.031–0.152)	0.003

Data fields with the ‘–’ symbol were not statistically significant

*ABS* Association of Breast Surgery, *ASTRO* American Society for Radiation Oncology, *CI* confidence interval, *DCIS* ductal carcinoma in situ, *ER* estrogen receptor, *HER2* human epidermal growth factor receptor 2, *PR* progesterone receptor, *SSO* Society of Surgical Oncology

Multivariate regression analysis was conducted to identify independent predictors of positive margin (Table 4). Applying ABS criteria to the dataset, younger age at index operation (0.033, 95% CI 0.006–0.060; *p *= 0.017), lower specimen weight (0.045, 95% CI 0.020–0.069; *p *< 0.001), multifocal disease (0.256, 95% CI 0.137–0.376; *p *< 0.001), LVI (0.138, 95% CI 0.068–0.208; *p *< 0.001), DCIS with comedonecrosis (0.113, 95% CI 0.040–0.185; *p *= 0.002) and DCIS present beyond IBC boundaries (0.115, 95% CI 0.042–0.188; *p *= 0.002) were observed to be independent clinic-pathological predictors of a positive margin. When applying SSO-ASTRO criteria, larger composite tumour size (0.009, 95% CI 0.005–0.013; *p *< 0.001) and DCIS with comedonecrosis (0.159, 95% CI 0.056–0.262; *p *= 0.002) were statistically significant independent predictors of a positive margin (Table 4).

**Table 4:** Multivariate analysis of association with positive margins comparing UK ABS guidelines with US SSO-ASTRO guidelines

Variable	UK ABS		US SSO-ASTRO	
	*β* coefficient (95% CI)	*p* value	*β* coefficient (95% CI)	*p* value
Younger age at index operation, years	0.033 (0.006–0.06)	0.017	–	–
Lower specimen weight, g	0.045 (0.02–0.069)	< 0.001	–	–
Multifocal disease	0.256 (0.137–0.376)	< 0.001	–	–
Larger composite size of tumour	–	–	0.009 (0.005–0.013)	< 0.001
DCIS present beyond invasive disease?	0.115 (0.042–0.188)	0.002	–	–
Lymphovascular invasion present	0.138 (0.068–0.208)	< 0.001	–	–
DCIS with comedonecrosis	0.113 (0.04–0.185)	0.002	0.159 (0.056–0.262)	0.002

Data fields with the ‘–’ symbol were not statistically significant

*ABS* Association of Breast Surgery, *ASTRO* American Society for Radiation Oncology, *CI* confidence interval, *DCIS* ductal carcinoma in situ, *SSO* Society of Surgical Oncology

### Reoperation Rates and Residual Disease

In this study, 239/909 (26.3%) patients underwent reoperation: 173/909 underwent re-excision surgery and the remaining 66/909 underwent a completion mastectomy. Residual disease was present in 90/239 (37.7%) patients who had further surgery. Regarding the 90 patients in whom residual disease was present, pure residual DCIS was identified in 60/90 (66.7%), pure residual IBC in 10/90 (11.1%) and combined DCIS and IBC in the remaining 20/90 (22.2%) (see Table 5).

**Table 5:** Summary of the data of patients who underwent reoperation, including their type of reoperation and residual histology

Reoperation details	No. of patients (%)
Reoperation	
Patients who underwent reoperation	239/909 (26.3)
No reoperation	670/909 (73.7)
Type of reoperation	
Re-excision of margins	173/239 (72.4)
Completion mastectomy	66/239 (27.6)
Residual disease	
Residual disease present	90/239 (37.7)
No residual disease present	149/239 (62.3)
Histology of residual disease	
Ductal	73/90 (81.1)
Lobular	14/90 (15.6)
Ductal and lobular	1/90 (1.1)
Other	2/90 (2.2)
Residual DCIS present	
Pure residual DCIS present	60/90 (66.7)
Mixed residual invasive disease and DCIS	20/90 (22.2)
No residual DCIS present	10/90 (11.1)

*DCIS* ductal carcinoma in situ

Univariate analysis demonstrated non-wire-guided surgery (i.e. palpable tumours) (0.863, 95% CI 0.008–0.057; *p *= 0.04) and tumours with no evidence of DCIS (0.397, 95% CI 0.082–0.852; *p *= 0.018) were significantly associated with no residual disease at reoperation.

Multivariate regression analysis calculated that tumours with no evidence of DCIS (0.438, 95% CI 0.004–0.069; *p *= 0.015) were statistically significant independent predictors of no residual disease in patients who underwent reoperation.

## Discussion

In this study, the RR of a positive margin with IBC + DCIS versus pure IBC was 8.76 (95% CI 6.64–11.56) and 8.44 (95% CI 6.57–10.84) (ABS and SSO-ASTRO criteria, respectively). These values were computed using verified and validated margin-level analysis and are significantly greater than previous analyses.^30–32,34,36–40^ Interestingly, a lack of DCIS is also an independent predictor of no residual disease at reoperation. Calculating the RR using patient-level data (primary tumour data compared with margin-specific data) align with figures found in the current literature.^5,21,49^ The RR of a positive margin in patients with DCIS in the primary tumour compared with pure invasive disease (ABS and SSO-ASTRO criteria, respectively) was RR 1.46 (95% CI 1.10–1.95) and RR 2.77 (95% CI 1.96–3.92).

To our knowledge, this is the largest and most comprehensive study to analyse margin-level data from over 5000 resection margins in 909 patients undergoing BCS, by a single reviewer. Margin-level details extracted included the histopathological findings at the margin and distance from the new resection margin alongside the histopathological diagnosis of the primary tumour in order to quantify risk of reoperation and calculate predictors. A margin-level approach, such as that used in the current study, is believed to be a more accurate representation of the risk related to DCIS.

One study reviewed data from 4118 patients with impalpable cancer and calculated the risk of reoperation was three times higher in patients with DCIS compared with IBC (odds ratio [OR] 3.82, 95% CI 3.19–4.58; *p *< 0.001); however, this was based on standardised data taken from the National Registry, and the diagnosis of DCIS versus IBC was obtained from the pathological diagnosis of the primary tumour rather than the findings at the margin.^21^ It is well known that DCIS may co-exist with IBC, evident in the diagnostic core and/or the final postoperative histopathology.^39^ In patients with core biopsy-proven IBC, the cause of a positive margin may be DCIS rather than invasive carcinoma, hence the potential to underestimate the burden of DCIS margin positivity. For example, in a large UK-based study of 55,297 patients undergoing BCS, those with in situ carcinoma in their primary diagnosis had increased risk of reoperation (OR 1.9, 90% CI 1.8–2).^5^ The Sloane Project, a prospective audit of UK screen-detected DCIS, calculated a 30% reoperation rate.^28^ This audit relied on UK screening units voluntarily submitting accurate data. Seventy percent of patients entered into this study had missing pathological or radiological data variables.^28^ Another factor that may add to the variation in reported positive margin rates is the lack of international consensus on the definition of a ‘positive margin’. Despite this, applying both UK and US guidelines to the data in the current study demonstrated similar results in significantly greater RR and OR compared with previous data.

Independent predictors of positive resection margins calculated in this study align with the current literature and include younger age at index operation,^30,50,51^ microcalcifications on preoperative mammography,^32–34,52^ lower specimen weight, tumour size,^33,36,38,40,51,52^ multifocal disease,^33,35^ LVI,^30,37,51^ DCIS with comedonecrosis^30,31,35,50,51^ and DCIS present beyond invasive disease margins.^31,36,52^ These predictors can be collated based on the weight in the ability to accurately predict the outcome of a positive margin. This risk stratification tool could be used alongside the toolbox created by the American Society of Breast Surgeons.^11^ This can be calculated at the preoperative multidisciplinary planning meeting to facilitate preoperative decision making and aid patient counselling and consent.

Reoperation delays adjuvant treatment, increases levels of wound infection and scarring, prolongs recovery and compounds emotional stress.^53^ In addition, there are direct implications on the health service, with additional patients on theatre lists, anaesthetic requirement, hospital bed days and further histopathological analysis.^54^ The findings in this study emphasise the urgent need for the development of a novel margin assessment tool that can accurately recognise DCIS in vivo. DCIS can co-exist with invasive cancer and the in situ component can extend beyond the invasive component. DCIS may be missed by existing margin detection tools due to limited spatial resolution or sampling coverage.^20^ In addition, the lack of a DCIS clinical correlate has demonstrated reduced effectiveness in intraoperative specimen imaging devices.^20^ However, there are more promising preliminary results with identifying DCIS with Raman Spectroscopy^20^ and Rapid Evaporative Ionisation Mass Spectrometry.^55^

This study has several limitations that should be acknowledged. This study, carried out using data from hospital databases, has a retrospective nature and was carried out at a single institution, and therefore may demonstrate institutional bias. There was no standardisation of surgeon, radiologist or histopathologist, which may have led to a degree of variability in reporting.

## Conclusion

The current study suggests the risk of a positive margin with IBC + DCIS is approximately ninefold the risk of an involved margin with pure IBC, regardless of whether UK or USA margin width criteria are applied. Surgeons should pay particularly close attention to demographic and clinicopathological factors that are associated with DCIS margin positivity, such as young age, multifocal disease, microcalcifications and comedonecrosis on the diagnostic core biopsy. It is critical that intraoperative margin assessment tools can accurately diagnose DCIS, to optimise oncological margin control in vivo.

### Supplementary Information

Below is the link to the electronic supplementary material.

Supplementary file1 (DOCX 18 kb)

## References

[1] National Cancer Registration and Analysis Service PHE. Chemotherapy, radiotherapy and surgical tumour resections in England 2013–2016. https://www.gov.uk/government/statistics/chemotherapy-radiotherapy-and-surgical-tumour-resections-in-england. Accessed 7 Jan 2022.

[2] National Collaborating Centre for Cancer. Breast cancer: diagnosis and treatment. https://www.nice.org.uk/guidance/cg81/evidence/needs-assessment-pdf-242246992. Accessed 7 Jan 2022.

[3] American Cancer Society. Breast cancer facts and figures 2017–2018. https://www.cancer.org/content/dam/cancer-org/research/cancer-facts-and-statistics/breast-cancer-facts-and-figures/breast-cancer-facts-and-figures-2017-2018.pdf. Accessed 7 Jan 2022.

[4] Getting it Right First Time, NHS England. Breast surgery: GIRFT programme national specialty report. https://future.nhs.uk/connect.ti/GIRFTNational/view?objectId=112160613. Accessed 7 Jan 2022.

[5] Jeevan R, Cromwell DA, Trivella M (2012). Reoperation rates after breast conserving surgery for breast cancer among women in England: retrospective study of hospital episode statistics. BMJ.

[6] Isaacs AJ, Gemignani ML, Pusic A, Sedrakyan A (2016). Association of breast conservation surgery for cancer with 90-day reoperation rates in New York State. JAMA Surg.

[7] Wilke LG, Czechura T, Wang C (2014). Repeat surgery after breast conservation for the treatment of stage 0 to II breast carcinoma: a report from the National Cancer Data Base, 2004–2010. JAMA Surg.

[8] de Camargo Cancela M, Comber H, Sharp L (2013). Hospital and surgeon caseload are associated with risk of re-operation following breast-conserving surgery. Breast Cancer Res Treat.

[9] van Leeuwen MT, Falster MO, Vajdic CM (2018). Reoperation after breast-conserving surgery for cancer in Australia: statewide cohort study of linked hospital data. BMJ.

[10] Spilsbury K, Semmens JB, Saunders CM, Hall SE, Holman C (2005). Subsequent surgery after initial breast conserving surgery: a population based study. ANZ J Surg.

[11] Landercasper J, Attai D, Atisha D (2015). Toolbox to reduce lumpectomy reoperations and improve cosmetic outcome in breast cancer patients: the American Society of Breast Surgeons Consensus Conference. Ann Surg Oncol.

[12] McCahill LE, Single RM, Bowles EJA (2012). Variability in reexcision following breast conservation surgery. JAMA.

[13] Houssami N, Macaskill P, Marinovich ML, Morrow M (2014). The association of surgical margins and local recurrence in women with early-stage invasive breast cancer treated with breast-conserving therapy: a meta-analysis. Ann Surg Oncol.

[14] Grant Y, Al-Khudairi R, St John E (2019). Patient-level costs in margin re-excision for breast-conserving surgery. Br J Surg.

[15] Engel J, Kerr J, Schlesinger-Raab A, Sauer H, Hölzel D (2004). Quality of life following breast-conserving therapy or mastectomy: results of a 5-year prospective study. Breast J.

[16] Gonzalez EA, Saltzstein EC, Riedner CS, Nelson BK (2003). Seroma formation following breast cancer surgery. Breast J.

[17] Al-Ghazal S, Fallowfield L, Blamey R (2000). Comparison of psychological aspects and patient satisfaction following breast conserving surgery, simple mastectomy and breast reconstruction. Eur J Cancer.

[18] Jagsi R, Li Y, Morrow M (2015). Patient-reported quality of life and satisfaction with cosmetic outcomes after breast conservation and mastectomy with and without reconstruction: results of a survey of breast cancer survivors. Ann Surg.

[19] Tang SSK, Kaptanis S, Haddow JB (2017). Current margin practice and effect on re-excision rates following the publication of the SSO-ASTRO consensus and ABS consensus guidelines: a national prospective study of 2858 women undergoing breast-conserving therapy in the UK and Ireland. Eur J Cancer.

[20] Dumitru D, Douek M, Benson JR (2018). Novel techniques for intraoperative assessment of margin involvement. Ecancermedicalscience.

[21] Langhans L, Jensen MB, Talman MLM, Vejborg I, Kroman N, Tvedskov TF (2017). Reoperation rates in ductal carcinoma in situ vs invasive breast cancer after wire-guided breast-conserving surgery. JAMA Surg.

[22] Pilewskie M, Morrow M (2018). Margins in breast cancer: how much is enough?. Cancer.

[23] Houvenaeghel G, Lambaudie E, Bannier M (2019). Positive or close margins: reoperation rate and second conservative resection or total mastectomy?. Cancer Manag Res.

[24] Harris EE, Schultz DJ, Jones HA, Solin LJ (2003). Factors associated with residual disease on re-excision in patients with ductal carcinoma in situ of the breast. Cancer J.

[25] Houvenaeghel G, Lambaudie E, Bannier M, Ribeiro S, Barrou J, Heinemann M (2018). Re-operation and mastectomy rates after breast conservative surgery for positive or close margins: a review. Clin Surg.

[26] Miligy IM, Toss MS, Khout H (2019). Surgical management of ductal carcinoma in situ of the breast: a large retrospective study from a single institution. Breast J.

[27] Thomas J, Hanby A, Pinder SE (2014). Adverse surgical outcomes in screen-detected ductal carcinoma in situ of the breast. Eur J Cancer.

[28] Thomas J, Evans A, Macartney J (2010). Radiological and pathological side estimations of pure ductal carcinoma in situ of the breast: a review of 2564 cases from the Sloane Project. Br J Cancer.

[29] Meijnen P, Oldenburg HS, Peterse JL, Bartelink H, Emiel JT (2008). Clinical outcome after selective treatment of patients diagnosed with ductal carcinoma in situ of the breast. Ann Surg Oncol.

[30] Aziz D, Rawlinson E, Narod SA (2006). The role of reexcision for positive margins in optimizing local disease control after breast-conserving surgery for cancer. Breast J.

[31] Miller AR, Brandao G, Prihoda TJ, Hill C, Cruz AB, Yeh IT (2004). Positive margins following surgical resection of breast carcinoma: analysis of pathologic correlates. J Surg Oncol.

[32] Mai KT, Chaudhuri M, Perkins DG, Mirsky D (2001). Resection margin status in lumpectomy specimens for duct carcinoma of the breast: correlation with core biopsy and mammographic findings. J Surg Oncol.

[33] Kurniawan ED, Wong MH, Windle I (2008). Predictors of surgical margin status in breast-conserving surgery within a breast screening program. Ann Surg Oncol.

[34] Shin H-C, Han W, Moon H-G (2012). Nomogram for predicting positive resection margins after breast-conserving surgery. Breast Cancer Res Treat.

[35] van Deurzen CH (2016). Predictors of surgical margin following breast-conserving surgery: a large population-based cohort study. Ann Surg Oncol.

[36] Vos E, Gaal J, Verhoef C, Brouwer K, Van Deurzen C, Koppert L (2017). Focally positive margins in breast conserving surgery: predictors, residual disease, and local recurrence. Eur J Surg Oncol.

[37] Smitt MC, Horst K (2007). Association of clinical and pathologic variables with lumpectomy surgical margin status after preoperative diagnosis or excisional biopsy of invasive breast cancer. Ann Surg Oncol.

[38] Chagpar AB, Martin RC, Hagendoorn LJ, Chao C, McMasters KM (2004). Lumpectomy margins are affected by tumor size and histologic subtype but not by biopsy technique. Am J Surg.

[39] Dzierzanowski M, Melville KA, Barnes PJ, MacIntosh RF, Caines JS, Porter GA (2005). Ductal carcinoma in situ in core biopsies containing invasive breast cancer: correlation with extensive intraductal component and lumpectomy margins. J Surg Oncol.

[40] Dillon MF, Hill AD, Quinn CM, McDermott EW, O’Higgins N (2006). A pathologic assessment of adequate margin status in breast-conserving therapy. Ann Surg Oncol.

[41] Volders J, Haloua M, Krekel N, Negenborn V, Barbé E, Sietses C (2016). Neoadjuvant chemotherapy in breast-conserving surgery: consequences on margin status and excision volumes: a nationwide pathology study. Eur J Surg Oncol.

[42] Association of Breast Surgery. ABS Consensus: Margn width in breast conservation surgery 2015. https://associationofbreastsurgery.org.uk/media/1418/abs-consensus-on-margin-width-in-breast-conservation-surgery.pdf. Accessed 7 Jan 2022.

[43] Tang S, Kaptanis S (2017). Variables associated with margin re-excision in breast conserving therapy (BCT)–results from the national margins audit. Eur J Surg Oncol.

[44] Morrow M, Van Zee KJ, Solin LJ (2016). Society of Surgical Oncology-American Society for Radiation Oncology-American Society of Clinical Oncology consensus guideline on margins for breast-conserving surgery with whole-breast irradiation in ductal carcinoma in situ. Ann Surg Oncol.

[45] Moran MS, Schnitt SJ, Giuliano AE (2014). SSO-ASTRO consensus guideline on margins for breast-conserving surgery with whole breast irradiation in stage I and II invasive breast cancer. Int J Radiat Oncol Biol Phys.

[46] D’Orsi CJ, Sickles EA, Mendelson EB, Morris EA, et al. ACR BI-RADS^®^ Atlas, breast imaging reporting and data system. Reston, VA: American College of Radiology; 2013.

[47] Patzer RE, Kaji AH, Fong Y (2021). TRIPOD reporting guidelines for diagnostic and prognostic studies. JAMA Surg.

[48] Collins GS, Reitsma JB, Altman DG, Moons KG (2015). Transparent reporting of a multivariable prediction model for individual prognosis or diagnosis (TRIPOD): the TRIPOD statement. J Br Surg.

[49] Jeevan R, Cromwell D, Trivella M, Lawrence G, Kearins O, Pereira J (2012). Reoperation rates after breast conserving surgery for breast cancer among women in England: retrospective study of hospital episode statistics. BMJ..

[50] Wazer DE, Schmidt-Ullrich RK, Ruthazer R (1999). The influence of age and extensive intraductal component histology upon breast lumpectomy margin assessment as a predictor of residual tumor. Int J Radiat Oncol Biol Phys.

[51] Singletary SE (2002). Surgical margins in patients with early-stage breast cancer treated with breast conservation therapy. Am J Surg.

[52] Ellbrant J, Gulis K, Plasgård E, Svensjö T, Bendahl P, Rydén L (2021). Validated prediction model for positive resection margins in breast-conserving surgery based exclusively on preoperative data. Br J Surg.

[53] Olsen MA, Nickel KB, Margenthaler JA (2015). Increased risk of surgical site infection among breast-conserving surgery re-excisions. Ann Surg Oncol.

[54] Yu J, Elmore LC, Cyr AE, Aft RL, Gillanders WE, Margenthaler JA (2017). Cost analysis of a surgical consensus guideline in breast-conserving surgery. J Am Coll Surg.

[55] St John ER, Balog J, McKenzie JS (2017). Rapid evaporative ionisation mass spectrometry of electrosurgical vapours for the identification of breast pathology: towards an intelligent knife for breast cancer surgery. Breast Cancer Res.

