# Supplementary material for: Clinicopathological Predictors of Positive Resection Margins in Breast-Conserving Surgery
Source: Ann Surg Oncol. 2024 Mar 23;31(6):3939–47. doi: 10.1245/s10434-024-15153-8 (PMC11076377; doi:10.1245/s10434-024-15153-8)
Supplement: Supplementary file 1 — Supplementary file1 (DOCX 18 kb) [file 10434_2024_15153_MOESM1_ESM.docx]

**Supplementary Table 1:** Summary of clinicopathological results based on patient demographics, pre-operative patient investigations and primary surgery details.

| **Characteristic** | **Number of patients, n/909 (%)** |
| --- | --- |
| **Age group:** |  |
| <40 | 31 (3.4) |
| 40 – 49 | 108 (11.9) |
| 50 – 59 | 287 (31.6) |
| 60 – 69 | 309 (34.0) |
| >70 | 174 (19.1) |
|  |  |
| **Referral route:** |  |
| Symptomatic | 313 (34.4) |
| Screening | 596 (65.6) |
|  |  |
| **Core biopsy histology:** |  |
| Pure invasive disease | 555 (61.1) |
| Pure in-situ disease | 210 (23.1) |
| Mixed invasive & in-situ disease | 144 (15.8) |
|  |  |
| Ductal carcinoma | 816 (89.8) |
| Lobular carcinoma | 50 (5.5) |
| Mucinous/papillary/tubular carcinoma | 43 (4.7) |
|  |  |
| **Pre-operative imaging results:** |  |
| Micro-calcifications present | 385 (42.4) |
| Micro-calcifications absent | 524 (57.6) |
|  |  |
| Dense breasts | 159 (17.5) |
| Breasts not dense | 750 (82.5) |
|  |  |
| MRI carried out | 180 (19.8) |
| MRI not carried out | 729 (80.2) |
|  |  |
| **Primary surgery details:** |  |
| Wire localisation performed | 762 (83.8) |
| Wire localisation not performed | 147 (16.2) |
|  |  |
| **Tumour type:** |  |
| Ductal | 772 (84.9) |
| Lobular | 62 (6.8) |
| Ductal & lobular | 9 (1) |
| Mucinous | 25 (2.8) |
| Papillary | 23 (2.5) |
| Tubular | 18 (2.0) |
|  |  |
| **DCIS presence:** |  |
| Pure DCIS | 190 (20.9) |
| DCIS with invasive disease | 537 (59.1) |
| Pure invasive disease | 182 (20.0) |
|  |  |
| **Tumour grade:** |  |
| In-situ | 190 (20.9) |
| 1 | 167 (18.4) |
| 2 | 446 (49.1) |
| 3 | 106 (11.7) |
|  |  |
| **Extension of DCIS beyond invasive disease:** |  |
| DCIS extends beyond invasive disease | 242 (26.6) |
| DCIS contained within invasive margins | 253 (27.8) |
| No invasive/DCIS component | 414 (45.5) |
|  |  |
| **Multifocality:** |  |
| Unifocal tumour present | 856 (94.2) |
| Multifocal tumour present | 53 (5.8) |
|  |  |
| **Hormone receptor status:** |  |
| ER positive | 678 (74.6) |
| ER negative | 42 (4.6) |
| ER status not tested | 189 (20.8) |
| PR positive | 577 (63.5) |
| PR negative | 143 (15.7) |
| PR status not tested | 189 (20.8) |
| HER2 positive | 65 (7.2) |
| HER2 negative | 655 (72.1) |
| HER2 status not tested | 189 (20.8) |
| Triple negative | 28 (3.1) |
|  |  |
| **Lymphovascular status:** |  |
| Lymphovascular invasion present | 200 (22.0) |
| Lymphovascular invasion not present | 709 (88) |
|  |  |
| **Comedonecrosis status:** |  |
| Comedonecrosis present | 208 (22.9) |
| Comedonecrosis not present | 701 (77.1) |
|  |  |
| **In-situ component status:** |  |
| In-situ component present | 726 (79.9) |
| In-situ component not present | 183 (20.1) |
|  |  |
| **Intra-operative shaves:** |  |
| Further shaves taken | 386 (42.5) |
| No further shaves taken | 523 (57.5) |

**Supplementary Table 2:** Overview of margin positivity data based on each individual margin.

| **Margins characteristic** | **Number of patients, n/909 (%)** | |
| --- | --- | --- |
|  | **UK ABS** | **US SSO-ASTRO** |
| **All margins negative** | 539 (59.3) | 507 (55.8) |
| **Positive margin(s) present** | 370 (40.7) | 402 (44.2) |
| **Positive radial margins** | **244 (26.8)** | **281 (30.9)** |
|  |  |  |
| **Anterior margin:** |  |  |
| Positive | 145 (16.0) | 160 (17.6) |
| Negative | 764 (84.0) | 749 (82.4) |
|  |  |  |
| **Posterior margin:** |  |  |
| Positive | 155 (17.1) | 175 (19.3) |
| Negative | 754 (82.9) | 734 (80.7) |
|  |  |  |
| **Medial margin:** |  |  |
| Positive | 92 (10.1) | 106 (11.7) |
| Negative | 817( 89.9) | 803 (88.3) |
|  |  |  |
| **Lateral margin:** |  |  |
| Positive | 98 (10.8) | 112 (12.3) |
| Negative | 811 (89.2) | 797 (87.7) |
|  |  |  |
| **Superior margin:** |  |  |
| Positive | 83 (9.1) | 108 (11.9) |
| Negative | 826 (90.9) | 801 (88.1) |
|  |  |  |
| **Inferior margin:** |  |  |
| Positive | 108 (11.9) | 113 (12.4) |
| Negative | 801 (88.1) | 796 (87.6) |
